# Antiarrhythmic Properties of Ranolazine: Inhibition of Atrial Fibrillation Associated TASK-1 Potassium Channels

**DOI:** 10.3389/fphar.2019.01367

**Published:** 2019-11-26

**Authors:** Antonius Ratte, Felix Wiedmann, Manuel Kraft, Hugo A. Katus, Constanze Schmidt

**Affiliations:** ^1^Department of Cardiology, University of Heidelberg, Heidelberg, Germany; ^2^DZHK (German Centre for Cardiovascular Research), partner site Heidelberg/Mannheim, University of Heidelberg, Heidelberg, Germany; ^3^HCR, Heidelberg Centre for Heart Rhythm Disorders, University of Heidelberg, Heidelberg, Germany

**Keywords:** atrial fibrillation, ranolazine, antiarrhythmic drugs, TASK-1, K2P3.1, KCNK3

## Abstract

**Background:** Atrial fibrillation (AF) is the most common sustained cardiac arrhythmia and one of the major causes of cardiovascular morbidity and mortality. Despite good progress within the past years, safe and effective treatment of AF remains an unmet clinical need. The anti-anginal agent ranolazine has been shown to exhibit antiarrhythmic properties *via* mainly late I_Na_ and I_Kr_ blockade. This results in prolongation of the atrial action potential duration (APD) and effective refractory period (ERP) with lower effect on ventricular electrophysiology. Furthermore, ranolazine has been shown to be effective in the treatment of AF. TASK-1 is a two-pore domain potassium (K_2P_) channel that shows nearly atrial specific expression within the human heart and has been found to be upregulated in AF, resulting in shortening the atrial APD in patients suffering from AF. We hypothesized that inhibition TASK-1 contributes to the observed electrophysiological and clinical effects of ranolazine.

**Methods:** We used *Xenopus laevis* oocytes and CHO-cells as heterologous expression systems for the study of TASK-1 inhibition by ranolazine and molecular drug docking simulations to investigate the ranolazine binding site and binding characteristics.

**Results:** Ranolazine acts as an inhibitor of TASK-1 potassium channels that inhibits TASK-1 currents with an IC_50_ of 30.6 ± 3.7 µM in mammalian cells and 198.4 ± 1.1 µM in *X. laevis* oocytes. TASK-1 inhibition by ranolazine is not frequency dependent but shows voltage dependency with a higher inhibitory potency at more depolarized membrane potentials. Ranolazine binds within the central cavity of the TASK-1 inner pore, at the bottom of the selectivity filter.

**Conclusions:** In this study, we show that ranolazine inhibits TASK-1 channels. We suggest that inhibition of TASK-1 may contribute to the observed antiarrhythmic effects of Ranolazine. This puts forward ranolazine as a prototype drug for the treatment of atrial arrhythmia because of its combined efficacy on atrial electrophysiology and lower risk for ventricular side effects.

## Introduction

Atrial fibrillation (AF) is a common cardiac rhythm disorder and one of the major causes of stroke, acute heart failure, sudden death, and cardiovascular morbidity (January et al., 2014). Despite good progress within the last years, safe and effective management of patients suffering from AF remains a major health issue, as current pharmacological, interventional or surgical therapeutic strategies are restricted by insufficient efficacy and often severe adverse effects (Kirchhof et al., 2016). Annual recurrence rates of AF after pharmacological cardioversion, for instance, range from 40 to 70% (Guerra et al., 2017). Furthermore, drug treatment with antiarrhythmic drugs (AADs) is often discontinued because of poor tolerability or adverse effects (Lafuente-Lafuente et al., 2015). For this reason, the development of safe and effective AADs for the treatment of AF is crucial (Dobrev and Nattel 2010).

Ranolazine is a drug originally introduced as an anti-anginal agent (Nash and Nash 2008) that has later been shown to exhibit antiarrhythmic properties *via* inhibition of different ion currents, especially the late phase of the inward sodium current (late I_Na_) and the rapidly activating delayed rectifier potassium current (I_Kr_) (Gupta et al., 2015). Because ranolazine predominantly prolongs atrial rather than ventricular action potential duration (APD) and effective refractory period (ERP) (Burashnikov et al., 2007; Antzelevitch and Burashnikov 2009), it appears to be particularly effective in AF (Guerra et al., 2017), thus far attributed to an atrial-selective sodium channel block (Sossalla et al., 2010; Antzelevitch et al., 2011).

TASK-1 (tandem of P domains in a weak inward rectifying K^+^ channel (TWIK)-related acid sensitive K^+^ channel 1; K_2P_3.1) is a member of the two-pore-domain potassium channel (K_2P_) family. This heterologous group comprises 15 members that share a unique structure of four transmembrane domains and two pore-forming loops per subunit which assemble as dimers (Goldstein et al., 2001). Regulated by a variety of physiological stimuli (extracellular pH, G-protein-mediated pathways, polyunsaturated fatty acids, temperature and mechanical stress) they provide a background “leak” potassium conductance modulating the cell’s resting membrane potential and cellular excitability (Feliciangeli et al., 2015). Their role in controlling cellular excitability predestines K_2P_ channels as potential players in diverse biological functions.

TASK-1 channels are widely expressed in various tissues, including the cerebral cortex (Vu et al., 2015), the brainstem retrotrapezoid (Mulkey et al., 2007) and pre-Botzinger regions (Koizumi et al., 2010), the carotid bodies (Buckler et al., 2000), hypoglossal and spinal cord motor neurons (Lazarenko et al., 2010), pulmonary artery smooth muscle (Olschewski et al., 2006), and the adrenal cortex (Czirjak and Enyedi 2002). They contribute in the regulation of oxygen sensing (Koizumi et al., 2010), endocrine secretion (Davies et al., 2008), auto-immune inflammation (Bittner et al., 2009), apoptosis (Lauritzen et al., 2003), and pulmonary blood pressure (Olschewski et al., 2006).

In the heart, TASK-1 is reported to modulate cardiac conduction, repolarization, and heart rate (Decher et al., 2011; Donner et al., 2011). Knockout or pharmacological inhibition of TASK-1 results in prolonged atrial APD and atrial ERP (Wirth et al., 2003; Putzke et al., 2007; Wirth et al., 2007; Decher et al., 2011; Skarsfeldt et al., 2016). Please note that some of the mentioned studies used inhibitors that are referred to as K_V_1.5 blockers (AVE0118 and AVE1231 (A293), developed by Sanofi, Paris, France). These inhibitors, however, later turned out to be much more potent TASK-1 blockers (Kiper et al., 2015). Wirth et al. (2007) demonstrated that TASK-1 blockade induced a prolongation of only atrial but not ventricular refractoriness and an associated inhibition of atrial vulnerability to arrhythmia. The prolongation of atrial refractoriness was even more pronounced in tachypacing induced AF and there were no effects on ECG intervals and ventricular repolarization. Within the human heart, TASK-1 has recently been shown to be predominantly expressed in the atrium as well, and TASK-1 inhibition results in prolonged APD on isolated human atrial cardiomyocytes (Schmidt et al., 2015). Because of enhanced TASK-1 currents under the condition of AF, the effect is even more pronounced and thus similar to the results obtained from large animal models. APD prolongation *via* TASK-1 blockade is expected to suppress AF and the ‘atrial selectivity’ of TASK-1 blockade by limiting the mode of action to atrial tissue, thereby reducing the risk of pro-arrhythmogenic effects in the ventricles, highlights the potential clinical significance of TASK-1 blockade for the treatment of AF in patients (Schmidt et al., 2017).

We hypothesized that ranolazine inhibits TASK-1 currents and that TASK-1 inhibition contributes to the observed antiarrhythmic effects of ranolazine. We chose *Xenopus laevis* oocytes and Chinese Hamster Ovary (CHO) cells as heterologous expression systems for detailed study of the biophysical characteristics of TASK-1 blockade by ranolazine. We further used *in silico* docking simulations and mutagenesis screen to explore structural determinants of TASK-1 blockade.

## Materials and Methods

### Molecular Biology

Complementary DNAs encoding human TWIK-1 (KCNK1; GenBank accession number NM_002245), TREK-1 (KCNK2; EF165334), TASK-1 (KCNK3; NM_002246), and TASK-3 (KCNK9; NM_016601) were kindly provided by Steve Goldstein (Chicago, IL, USA). Human TRESK cDNA (KCNK18; NM_181840) was obtained from C. Spencer Yost (San Francisco, CA, USA). Amplification of human TRAAK (KCNK4; EU978935), TASK-2 (KCNK5; EU978936), TWIK-2 (KCNK6; EU978937), TREK-2 (KCNK10; EU978939), THIK-1 (KCNK13; EU978942), TALK-1 (KCNK16; EU978943), and TALK-2 (KCNK17; EU978944) was previously described (Gierten et al., 2008). For *in vitro* transcription, cDNAs were subcloned into pRAT, a dual-purpose expression vector containing a cytomegalovirus promotor for mammalian expression and a T7 promotor for copy (c)RNA synthesis. All TASK-1 mutants reported in this study were generated using the QuikChange II Site-Directed Mutagenesis Kit (Agilent, Santa Clara, CA, USA) and synthetic mutant oligonucleotide primers. Sequences of all plasmid constructs were verified by DNA sequencing (GATC Biotech, Konstanz, Germany). After vector linearization with XbaI (New England Biolabs, Ipswich, MA, USA), plasmids were transcribed using the T7 mMessage mMachine kit (Thermo Fisher Scientific Inc., Waltham, MA, USA). Integrity of cRNA transcripts was assessed by agarose gel electrophoresis and cRNA concentrations were determined using Nanodrop spectrophotometry (ND-1000, peqLab Biotechnology GmbH, Erlangen, Germany).

### Cell Culture

Chinese hamster ovary (CHO) cells (CLS Cat# 603479/p746_CHO, RRID : CVCL_0213) were cultured in Dulbecco’s modified Eagle’s medium (DMEM, Thermo Fisher Scientific Inc., Waltham, MA, USA) supplemented with 10% fetal bovine serum (FBS, Thermo Fisher Scientific Inc., Waltham, MA, USA), 100 U/ml penicillin G sodium and 100 µg/ml streptomycin sulphate in an atmosphere of 95% humidified air and 5% CO_2_ at 37 °C. Cells were passaged regularly and seeded on glass cover slips prior to treatment. Transient transfections of CHO cells (passage 10–20) were performed using FuGENE HD transfection reagent (Promega, Madison, WI, USA) according to the manufacturer’s instructions. Cells were co-transfected with 2.2 µg pRAT-hTASK-1 plasmid DNA and 1.1 µg green fluorescent protein (GFP) plasmid DNA (pEGFP-N1; Clontech Laboratories, Mountain View, CA, USA) per 35 mm petri dish. Patch Clamp recordings were performed 24–36 h after transfection only on green fluorescent cells.

### *X. Laevis* Oocyte Preparation

This study was carried out in accordance with the directive 2010/63/EU of the European Parliament, and the current version of the German Law on the Protection of Animals. Approval for experiments involving *X. laevis* was granted by Regierungspräsidium Karlsruhe (institutional approval numbers A-38/11 and G-221/12). For detailed information on oocyte preparation please refer to the supplementary methods.

### Electrophysiology

Two-electrode voltage clamp (TEVC) recordings from *X. laevis* oocytes were performed one to three days after cRNA injection using an OC-725C Oocyte Clamp amplifier (Warner Instruments, Hamden, CT, USA), a Digidata 1322A Series (Axon Instruments, Foster City, CA, USA) and pClamp 10 software (Molecular Devices, San José, CA, USA). Current recordings from CHO cells were carried out using the whole-cell patch clamp technique with an Axopatch 200B amplifier (Axon Instruments, Foster City, CA, USA), an Axon Digidata 1550B series (Axon Instruments, Foster City, CA, USA), and pClamp 10 software (Molecular Devices, San José, CA, USA). For detailed information about solutions and test protocols please refer to the supplementary methods.

### Molecular Modelling and *In Silico* Drug Docking

Because the crystal structure of the human TASK-1 channel was not revealed yet, we built homology models using the SWISS-MODEL platform (Benkert et al., 2011; Bertoni et al., 2017; Waterhouse et al., 2018). Four models were generated based on the structures of TWIK-1 (protein data bank (PDB) ID: 3UMK) (Miller and Long 2012), TREK-1 (PDB ID: 6CQ6) (Lolicato et al., 2017), TREK-2 (PDB ID: 4XDL) (Dong et al., 2015) and TRAAK (PDB ID: 4RUE) (Lolicato et al., 2014). Quality assessment of the newly generated TASK-1 homology models was performed using MolProbidity (Davis et al., 2007).

Molecular docking calculations were performed using AutoDock Vina (Trott and Olson 2010). Analysis of protein–ligand interactions was performed using PLIP (Salentin et al., 2015). Three-dimensional visualizations of *in silico* simulations and dockings were generated with PyMOL 1.8 (PyMOL Molecular Graphics System, Schrödinger, LLC, New York, NY, USA).

### Data Analysis and Statistics

PCLAMP (Molecular Devices, San José, CA, USA), GraphPad Prism 6 (GraphPad Software Inc., La Jolla, CA, USA) and Microsoft Excel (Microsoft, Redmond, WA, USA) software was used for data acquisition and analysis. The concentration required for 50% block of current (half-maximal inhibitory concentration (IC_50_)) was calculated from Hill plots using Prism 6 (GraphPad). Data are expressed as the mean ± standard error of the mean (SEM) unless stated otherwise. Paired and unpaired t-tests (two-tailed tests) were applied to compare the statistical significance of the results. P < 0.05 was considered statistically significant. Multiple comparisons were performed using one-way analysis of variance (ANOVA). If the hypothesis of equal means could be rejected, post-hoc comparisons of groups were made and the probability values were adjusted for multiple comparisons using the Bonferroni correction.

### Materials

Ranolazine-dihydrochloride was obtained from Selleck Chemicals (Munich, Germany) and dissolved in water to a 100 mM stock solution. Aliquots of the stock solution were stored at −20 °C and diluted to the desired concentration with the bath solution on the day of experiments. The dilution of Ranolazine did not affect the pH of the bath solution.

## Results

### Ranolazine Inhibits Human Task-1 Channels

**Figure 1:**
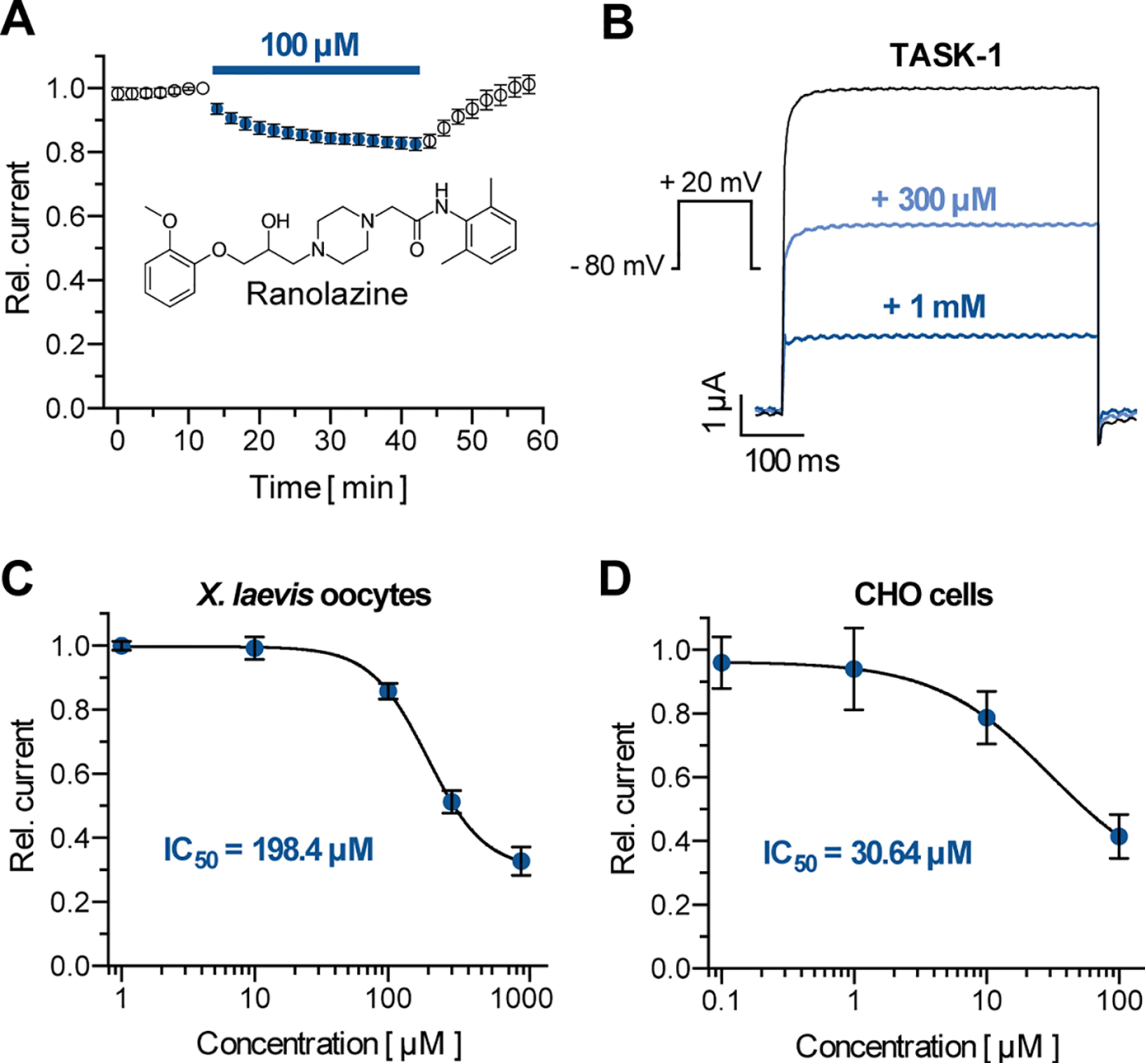
Effect of ranolazine on TASK-1 current. **(A)** Time course of TASK-1 current reduction during ranolazine application (n = 6); currents are normalized to their respective value before ranolazine application after a stabilization period with no significant amplitude changes. **(B)** Representative TASK-1 current recordings evoked by applying a test pulse from −80 mV to +20 mV under control conditions and after 30 min incubation with 300 µM and 1 mM ranolazine. **(C)** Dose–response curve of TASK-1 inhibition by ranolazine in *X. laevis* oocytes. Currents are normalized to their respective values under control conditions (n = 5 – 10). **(D)** Dose–response curve of TASK-1 inhibition by ranolazine in CHO cells. Currents are normalized to their respective values under control conditions (n = 5). Data are given as mean ± SEM.

To probe the inhibitory effects of ranolazine on human TASK-1 channels, TASK-1 was expressed in *X. laevis* oocytes. After a stabilization period with no significant current amplitude changes (15 min) ranolazine was administered for 30 min. Application of 100 µM ranolazine inhibited TASK-1 currents by 17.4 ± 2% (**Figure 1A**, n = 6, p = 0.018). Inhibitory effects were completely reversible. Current levels reached 100 ± 30% (n = 6) after 15 min washout period. TASK-1 channels were blocked with an IC_50_ of 30.6 ± 3.7 µM in CHO cells, and 198.4 ± 1.1 µM in oocytes, analyzed at +20 mV (**Figures 1C, D**). This 6.5-fold difference is consistent with previous findings that IC_50_ values determined in oocytes are mostly higher than those determined in mammalian cells (Streit et al., 2011; Schmidt et al., 2013). The maximum inhibition of 67.3 ± 4.5% in oocytes and 58.5 ± 6.9% in CHO cells was achieved with ranolazine concentrations of 1 mM and 100 µM, respectively. Administration of higher concentrations (3 mM in oocytes, 300 µM in CHO cells) led to cell instability and death. We used a concentration of 300 µM ranolazine for further experiments in *X. laevis* oocytes in order to achieve 50% current inhibition.

### Biophysical Characteristics of TASK-1 Channel Blockade by Ranolazine

**Figure 2:**
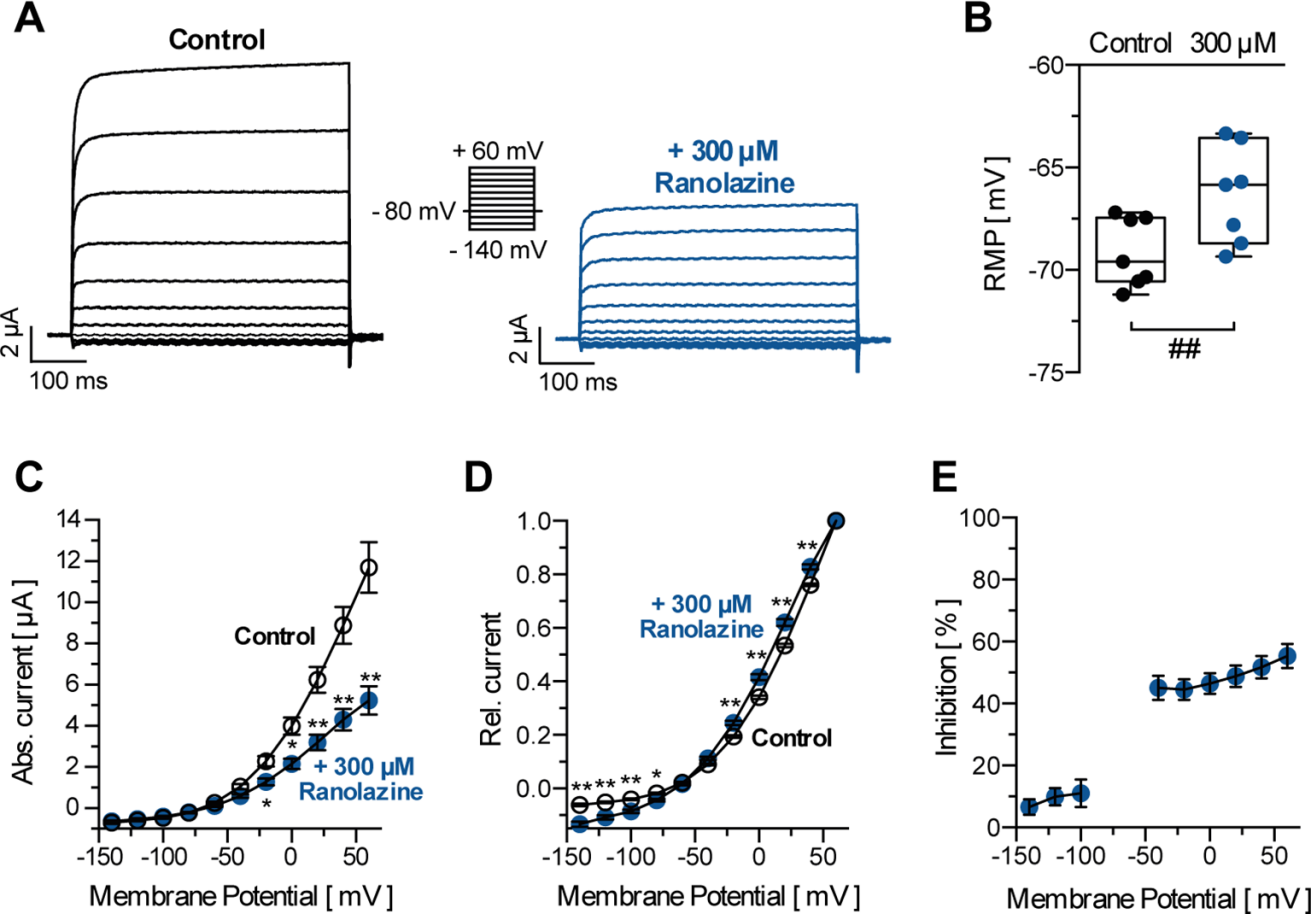
**(A)** Representative TASK-1 current recordings evoked by applying the displayed pulse protocol under control conditions and after 30 min incubation with ranolazine (300 µM). **(B)** Resting membrane potentials (RMP) in *X. laevis* oocytes before and after 30 min incubation with 300 µM ranolazine (n = 7). Boxes indicate the first and third quartile, whiskers indicate the minimum and maximum, and bands inside the boxes indicate the median; ##p < 0.01, paired t-test. **(C)** Activation curve of TASK-1 current; amplitudes are plotted against the respective test potential under control conditions and after 30 min incubation with ranolazine (n = 10); *p < 0.05, **p < 0.01, unpaired t-test vs. control conditions. **(D)** Amplitudes are normalized to the maximum current amplitude at +60 mV (n = 10); *p < 0.05, **p < 0.01, unpaired t-test vs. control conditions. **(E)** Dependency of TASK-1 current inhibition by ranolazine on membrane potential (n = 10). Data are given as mean ± SEM.

TASK-1 channels show electrophysiologic characteristics that are typical for most K_2P_ channels. They exhibit strong outward rectification with minimal inward current (0.69 ± 0.07 µA at −140 mV, n = 10) and a higher open probability at more depolarized membrane potentials (**Figures 2A, C, D**). **Figure 2A** illustrates representative current recordings of TASK-1 under control conditions and after application of 300 µM ranolazine for 30 min. Ranolazine (300 µM) inhibited TASK-1 currents by 48.79 ± 3.52% (analyzed at the end of the +20 mV test pulse, n = 10, p = 0.0004). Ranolazine also altered the resting membrane potential (RMP) from −66.57 ± 2.62 mV (control conditions, n = 10) to −63.55 ± 2.89 mV (300 µM ranolazine, n = 10, p = 0.0012, **Figure 2B**). Inhibitory effects of ranolazine showed voltage dependency, with less inhibition of inward currents (6.57 ± 2.55% to 11.07 ± 4.51% inhibition at voltages between −140 and −100 mV, n = 10) as compared to outward currents (44.56 ± 3.42% to 55.3 ± 3.87% inhibition at voltages between −40 and +60 mV, n = 10, **Figure 2E**). This resulted in an altered current-voltage (I-V) relationship under ranolazine treatment (**Figure 2D**).

**Figure 3:**
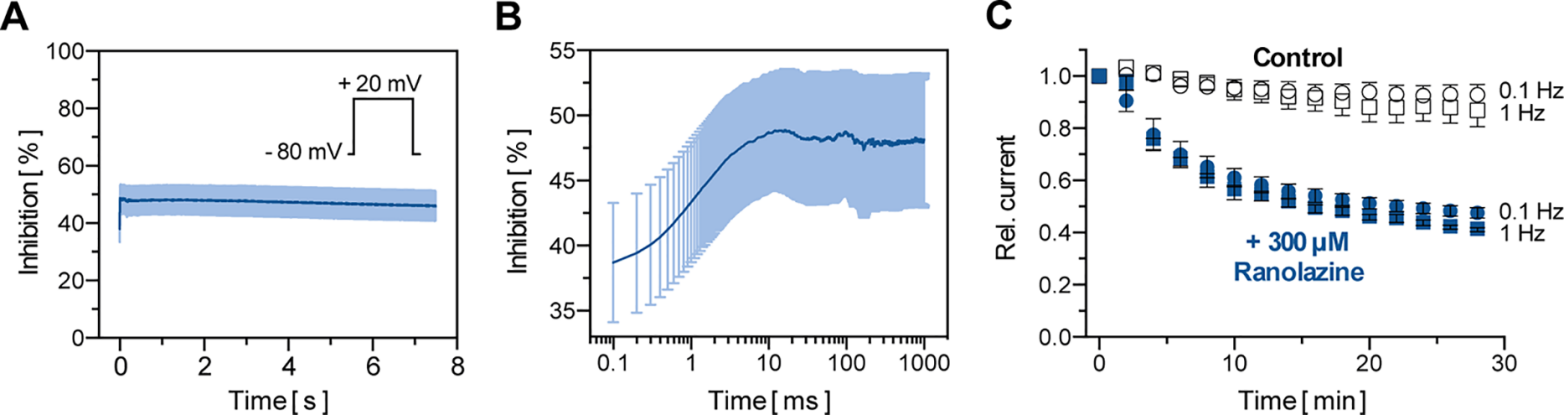
Biophysical properties of TASK-1 blockade by ranolazine. **(A)** The inhibition of TASK-1 current is displayed over a 7.5 s test pulse from −80 mV to +20 mV (n = 6). **(B)** Representation of the first 1000 ms of (A) on a logarithmic time scale (n = 6). **(C)** Frequency dependency of TASK-1 blockade by ranolazine; current amplitudes obtained during 30 min stimulation from −80 mV to +20 mV at stimulation rates of 0.1 Hz and 1 Hz are displayed under control conditions and during administration of 300 µM Ranolazine (n = 5–6). Currents are normalized to their respective values after a stabilization period. Data are given as mean ± SEM.

Macroscopic TASK-1 currents in heterologous expression systems activate in two phases. Currents activate quickly to approximately 85% of their respective maximum amplitude within the first 50 ms, followed by a markedly slower additional activation time course. Thus, TASK-1 currents may be divided into an instantaneous and a sustained current component. **Figures 3A, B** illustrate the inhibition of TASK-1 currents by ranolazine during a 7.5 s test pulse from −80 mV to +20 mV (n = 6). The maximum inhibition of 48.67 ± 4.45% was reached after 10 ms and remained unchanged over the 7.5 s test pulse. Additionally, TASK-1 inhibition by ranolazine did not show frequency dependency (**Figure 3C**). The slight difference between 0.1 Hz and 1 Hz may be attributed to higher cell instability at higher stimulation frequencies, as this difference was also observed under control conditions in absence of ranolazine.

### Effects of Ranolazine on Other Human Two-Pore Domain Potassium (K_2P_) Channels

**Figure 4:**
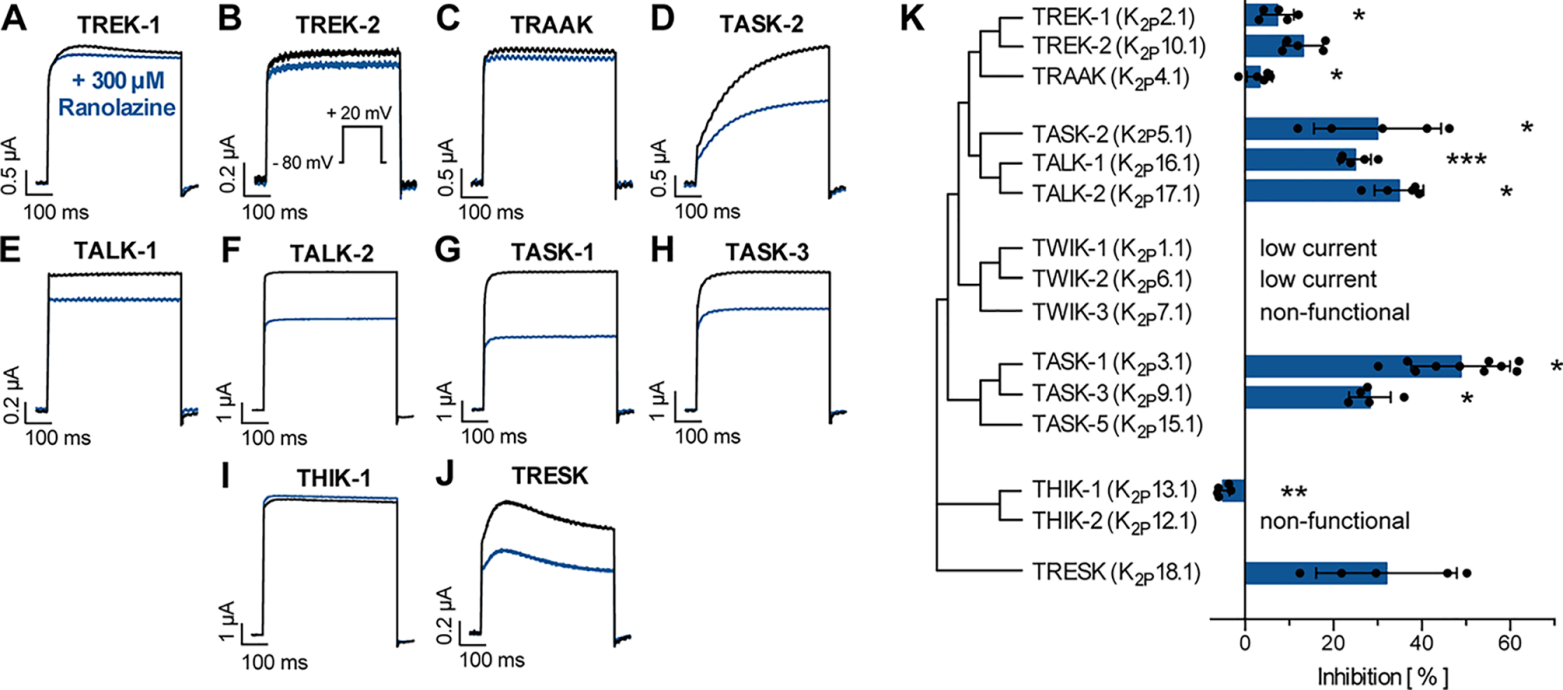
Effect of ranolazine on different members of the K_2P_ channel family. **(A–J)** Representative current recordings evoked by a test pulse from −80 mV to +20 mV under control conditions and after 30 min incubation ranolazine. **(K)** Current inhibition by 300 µM ranolazine is displayed for the different K_2P_ channel family members. Data are given as mean ± SEM. *p < 0.05, **p < 0.01, ***p < 0.001, paired t-test vs. control measurements.

We further tested inhibitory effects of ranolazine on other channels within the K_2P_ channel family using a similar approach as reported in **Figure 1A**. Ranolazine (300 µM) was administered after a stabilization period with no significant current amplitude changes (15–20 min). Inhibition of ion currents was quantified after 30 min of ranolazine administration (**Figure 4**). Ranolazine showed small inhibitory effects on TREK-1 (7.35 ± 1.66%, n = 5, p = 0.026) and TRAAK (3.32 ± 1.29%, n = 5, p = 0.045), and more pronounced inhibition of TASK-2 (30.02 ± 6.42%, n = 5, p = 0.037), TALK-1 (23.04 ± 3.06%, n = 5, p = 0.0003), TALK-2 (34.88 ± 2.47%, n = 5, p = 0.024) and TASK-3 currents (28.28 ± 2.1%, n = 5, p = 0.028). Effects on TREK-2 and TRESK were not significant. Ranolazine treatment of THIK-1 lead to slightly enhanced currents (+4.98 ± 0.66%, n = 5, p = 0.002). Taken together, ranolazine favorably inhibits K_2P_ channels that are acid sensitive (TASK-1, TASK-2, TASK-3) or alkaline sensitive (TALK-1, TALK-2).

### Protein–Ligand Interaction Profile of Ranolazine in the TASK-1 Inner Pore

**Figure 5:**
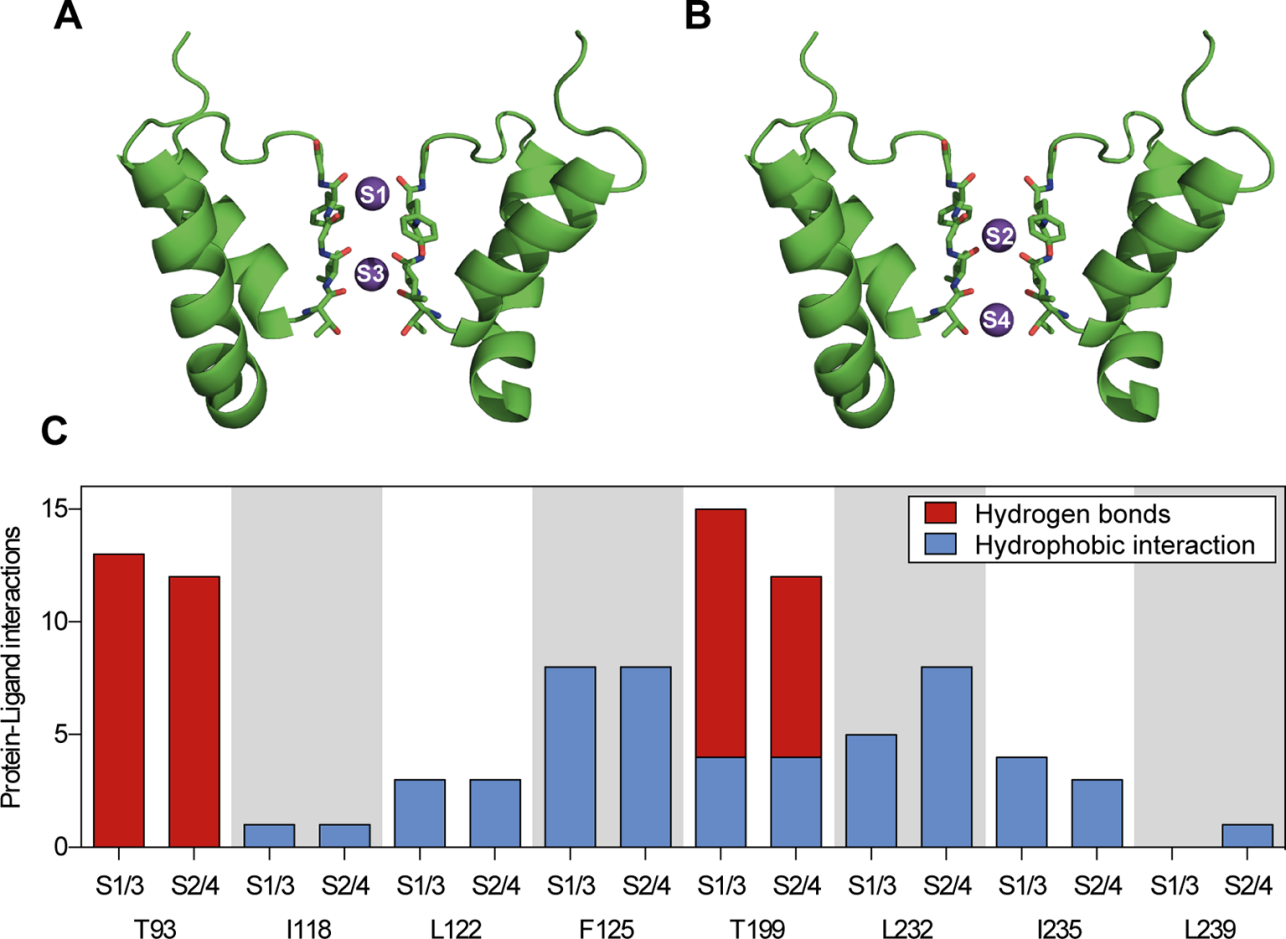
Summary of protein–ligand interactions of all calculated docking poses of ranolazine at the inner pore of the wild type (WT) TASK-1 model (T16cq6). **(A**, **B)** Docking calculations have been performed with potassium ions either at positions S1 and S3, or at positions S2 and S4. **(C)** Each calculation yielded 10 ranked docking poses, resulting in a total of 20 poses. Protein–ligand interactions have been analyzed using PLIP (Salentin et al., 2015). The amount and character of the interactions is displayed for the individual amino acid residues that interact with ranolazine.

TASK-1 potassium channels contain four ion binding positions (S1–S4) within the selectivity filter. For *in silico* docking calculations potassium ions were either positioned at S1 and S3 or at S2 and S4 (**Figures 5A, B**). Ten ranked docking poses were calculated for each configuration. The amount and character of the protein–ligand interactions of all docking poses is summarized in **Figure 5C** and displayed separately for the different ion binding configurations. Ranolazine is suggested to form hydrogen bonds with the threonine residues on position T93 and T199 and additional hydrophobic interactions with other pore lining residues. Different ion occupations within the selectivity filter only resulted in a slightly altered interaction profile with fewer hydrogen bonds formed by ranolazine and the threonine residues T93 and T199, and more hydrophobic interactions at position L232.

### Ranolazine Inhibition Depends on Amino Acid Residues Located at the Inner Pore of TASK-1

**Figure 6:**
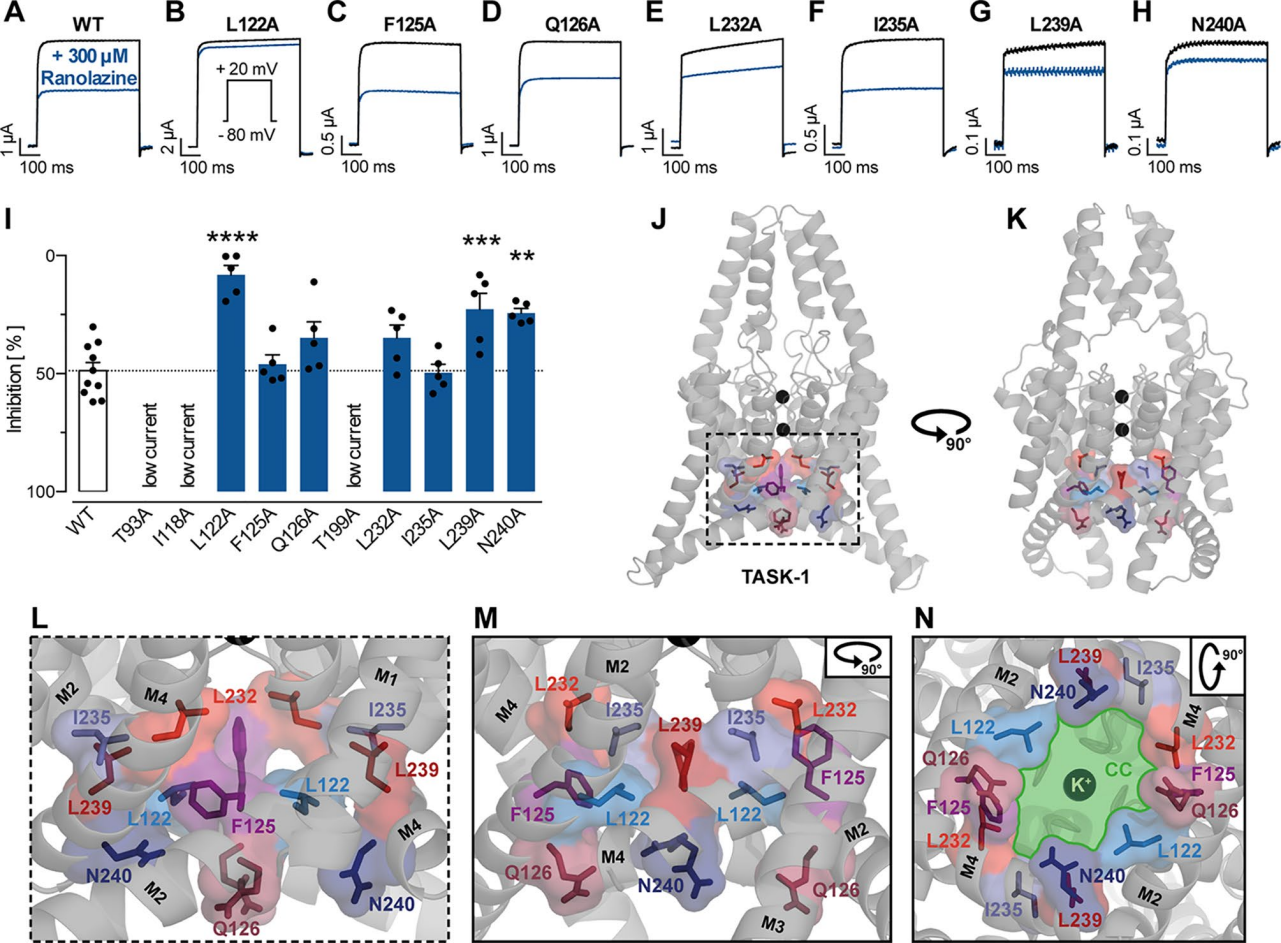
Effect of ranolazine on TASK-1 pore mutants. **(A–H)** Representative current recordings evoked by a test pulse from −80 mV to +20 mV under control conditions and after 30 min incubation with ranolazine. **(I)** Current inhibition by 300 µM ranolazine on the different TASK-1 pore mutants, **p < 0.01, ***p < 0.001, ****p < 0.0001, one-way ANOVA and post-hoc Bonferroni’s multiple comparisons test of WT vs. the respective mutant. **(J**, **K)** TASK-1 homology model based on TREK-1 illustrating the location of amino acid residues included in the mutagenesis screen. **(L)** Zoom into the inner pore region. **(M)** Lateral view as in panel **(L)** but from a different angle [as in panel **(K)**]. **(N)** View from inside the cell into the central cavity (CC) of the inner pore. Note how the displayed residues line the central cavity (illustrated in green) of the TASK-1 inner pore.

We further investigated the binding site of ranolazine within the TASK-1 inner pore, by individually mutating amino acids that were either predicted to contribute to ranolazine binding in molecular docking simulations, or have been identified as binding site for the high affinity blockers S20951 (A1899) and AVE1231 (A293) (Streit et al., 2011; Wiedmann et al., 2019). Variants T93A, I118A and T199A produced very low current amplitudes that did not allow reasonable pharmacology testing. Inhibitory effects of ranolazine on the functionally active TASK-1 mutants were quantified after 30 min ranolazine incubation at the end of a 500 ms test pulse from −80 mV to +20 mV. **Figure 6 A-H** indicates representative current recordings evoked by a test pulse from -80 mV to +20 mV under control conditions and after 30 min incubation with ranolazine. **Figure 6I** summarizes the current inhibition by 300 µM ranolazine on the different TASK-1 pore mutants. Inhibition of TASK-1 by 300 µM ranolazine was significantly reduced in channel variants L122A (from 48.79 ± 3.52% in wild type (WT) channels to 8.1 ± 3.94% in the mutant variant, n = 5, p < 0.0001), L239A (reduced to 22.69 ± 6.73%, n = 5, p = 0.0007), and N240A (reduced to 24.29 ± 1.92%, n = 5, p = 0.0016). The inhibitory potency of ranolazine remained unchanged in channel variants F125A, Q126A, L232A, and I235A. Note that the amino acids that were included in our mutagenesis screen all line the central cavity of the TASK-1 inner pore (**Figure 6 J-N**). 

**Figure 7:**
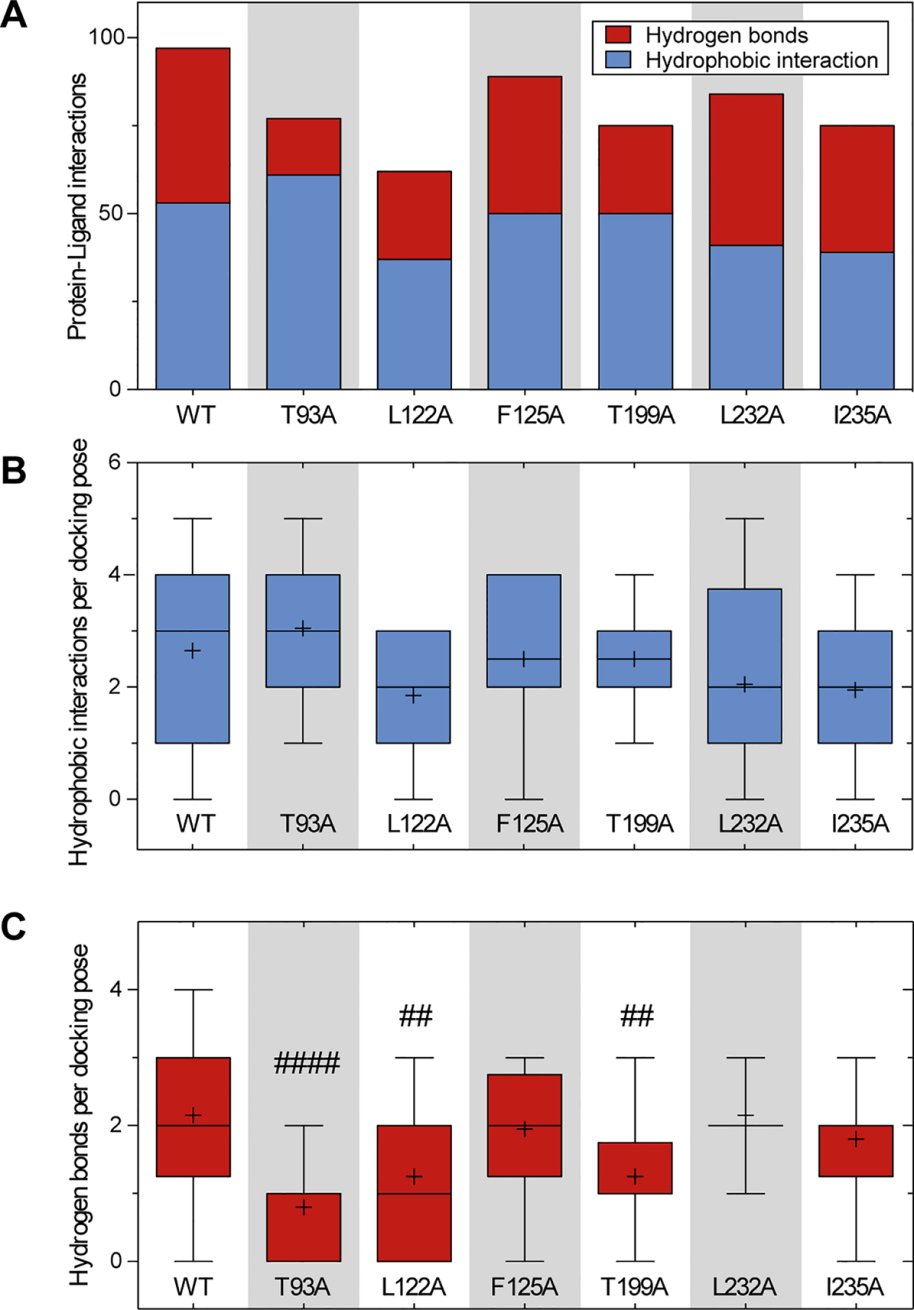
Comparison of the amount of protein–ligand interactions for the docking poses of WT and mutant channels. **(A)** Total amount and character of protein–ligand interactions of all 20 docking poses for the respective channel variant. **(B**, **C)** Boxplots indicating the amount of hydrophobic interactions **(B)** and hydrogen bonds **(C)** per docking pose. Boxes indicate the first and third quartile, whiskers indicate the minimum and maximum, bands inside the boxes indicate the median, and + indicates the mean. ##p < 0.01, ####p < 0.0001vs. WT. Note that variant L122A that has been identified as being most relevant for ranolazine binding in the experimental data, also forms the fewest interactions in the *in silico* simulations. More detailed information on the interaction profiles of ranolazine and the individual mutant variants can be found in **Supplementary Figure S3**.

We further performed *in silico* docking simulations on TASK-1 alanine mutants at positions T93, L122, F125, T199, L232, and I235 that had been identified as the most relevant residues for ranolazine binding in the initial WT docking simulation. The results (summarized in **Figure 7**) suggest, that in channel variants F125A, L232A and I235A, ranolazine was still able to bind and form protein–ligand interactions at the altered binding site. In channel variant L122A the ability to form protein–ligand interactions was impaired; especially the number of relatively stable hydrogen bonds was significantly reduced compared to WT. More detailed information on the individual interaction profiles of ranolazine and the different TASK-1 mutant variants can be found in the supplementary material (**Supplementary Figure S3**).

**Figure 8:**
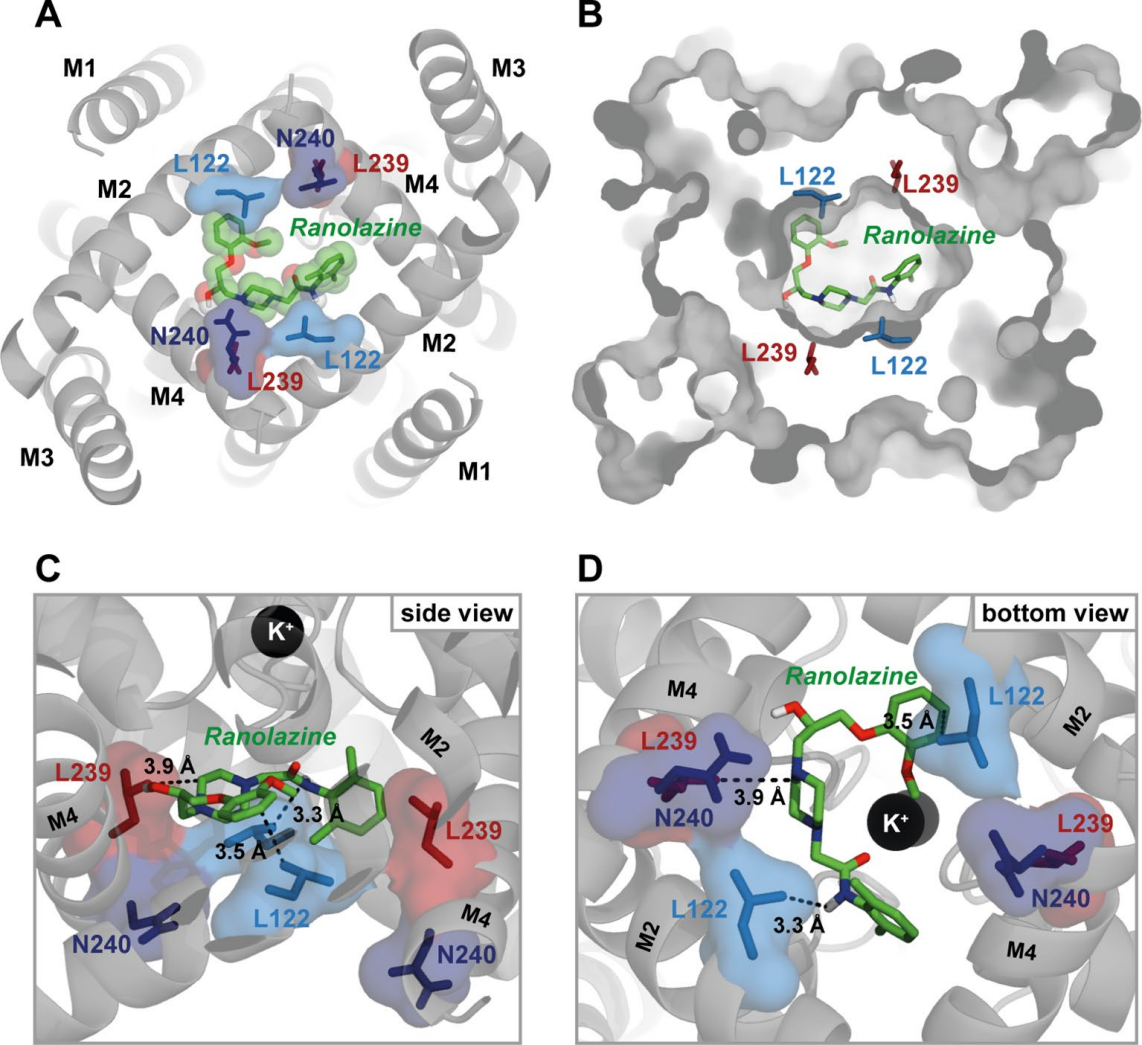
Ranolazine binds to the central cavity of the inner pore. **(A)** View from inside the cell into the central cavity. Ranolazine binds in close proximity to L122 and L239 occluding the lumen of the inner pore. **(B)** Same view as in **(A)** but with TASK-1 surface representation illustrating how ranolazine embeds within the inner pore. **(C)** Zoom into the central cavity illustrating the binding mode and the distance to the residues identified as binding site in the mutagenesis screen. Note that ranolazine does not directly interact with N240. **(D)** Similar zoom as in panel **(C)** but from inside the cell. Note that ranolazine interacts with L122 residues from both monomers.

**Figure 8** illustrates the best ranked ranolazine docking pose for WT TASK-1 calculated by AutoDock Vina. Ranolazine is suggested to build a flat layer that binds within the central cavity of the TASK-1 inner pore at the bottom of the selectivity filter occluding the lumen. Ranolazine is located in close proximity (< 4 Å) to amino acid residues L122 and L239 that have also been shown to be relevant for drug binding in the mutagenesis screen. However, N240 is not predicted to contribute to direct drug binding, although the mutant N240A showed reduced ranolazine inhibition.

In the illustrated docking pose ranolazine is also predicted to interact with residues F125 and L232, that showed no alteration of inhibition in the experimental data. Ranolazine is further predicted to form hydrogen bonds with residues T93 and T199. The relevance of T93 and T199 could not be validated experimentally due to too small current amplitudes of channel variants T93A and T199A.

## Discussion

The development of safe and effective AADs for the treatment of AF is a major clinical challenge (Dobrev and Nattel 2010). Within the past years ranolazine has been identified to deploy potent antiarrhythmic properties and to be effective in the treatment of AF (Guerra et al., 2017). Here we show that ranolazine acts as an inhibitor of the atrial selective TASK-1 potassium channel.

For the prevention of post-operative atrial fibrillation (POAF), ranolazine has been shown to be even more effective than amiodarone (Miles et al., 2011). In this retrospective trial there was no difference in the incidence of adverse events. Ranolazine, however, is considered to be substantially safer in use than amiodarone or other AADs, because it has less proarrhythmic effects on the ventricles (Gupta et al., 2015). This seems to be due to a differential impact on atrial and ventricular cardiomyocytes (Antzelevitch et al., 2011). Burashnikov et al. (2007) noticed that ranolazine was able to prolong the atrial APD and ERP in canine cardiomyocytes with only little effect on the ventricular action potential (AP). They attributed this effect to the inhibition of peak I_Na_ only in atrial but not ventricular cardiomyocytes and therefore proposed atrial-selective sodium channel blockade by ranolazine as a strategy for the treatment of atrial fibrillation (Burashnikov et al., 2007). The mechanism of the atrial-selective block of Na^+^ channels has been explained primarily by the rapid dissociation kinetics of ranolazine, a more negative half-inactivation voltage (V_0.5_) in atrial cells than in ventricular cells and a more depolarized RMP in atrial cells. Unblocking of sodium channels is commonly associated with the resting state of the sodium channel (Carmeliet and Mubagwa 1998). Because of a more negative half-inactivation voltage (V_0.5_) in atrial cells than in ventricular cells, a greater fraction of atrial sodium channels would remain in the inactivated state. Therefore the proportion of time that channels are in the resting state would be reduced and hence the dissociation of ranolazine would be slower. Additionally, the availability of sodium channels and the number of channels in the resting state would be further reduced by a more depolarized RMP in atrial cells and the recovery of sodium channels from the inactivated to the resting state is reported to be slower in atrial cells (Antzelevitch and Burashnikov 2009; Nesterenko et al., 2011). Literature regarding a different V_0.5_ in atrial vs. ventricular cardiomyocytes, however, is inconsistent (Sakakibara et al., 1992; Sakakibara et al., 1993; Hiroe et al., 1997; Li et al., 2002). Caves et al. (2017) could recently reproduce atrial selectivity of I_Na_ block by ranolazine in rabbit, providing further evidence that this might be a key element in the atrial-ventricular differences in the action of ranolazine (Caves et al., 2017).

Another explanation for the differential impact on APD prolongation in atrial and ventricular cardiomyocytes lies in the differences of AP configuration between the two cell types that have direct influence on the net effect of simultaneous late I_Na_ and I_Kr_ blockade. Ventricular APs have a more prominent plateau phase than atrial APs. This suggest that there is a smaller late I_Na_ in atrial cells that may result in a shift towards a greater AP prolonging effect of I_Kr_ blockade in atrial cells, whereas in ventricular cells the inhibition of a larger late I_Na_ may offset the I_Kr_ blockade (Du et al., 2014).

We propose that inhibition of TASK-1 potassium channels may contribute to the observed electrophysiological effects of ranolazine. Inhibition of TASK-1 by specific inhibitors has been described to prolong atrial APD and ERP before (Wirth et al., 2003; Wirth et al., 2007; Decher et al., 2011; Donner et al., 2011; Skarsfeldt et al., 2016). Furthermore, the nearly atrial specific expression of TASK-1 could, at least in part, explain the differential behavior of ranolazine between atrial and ventricular tissue (Limberg et al., 2011; Schmidt et al., 2015; Schmidt et al., 2017). The IC_50_ of TASK-1 inhibition by ranolazine is determined at 30.64 µM in mammalian cells which is slightly beyond therapeutic free plasma levels of 2–13.35 µM (Jerling 2006; Undrovinas et al., 2006; EMA 2009; Antzelevitch et al., 2011). Considering that ranolazine has its highest antiarrhythmic potency when administered in high doses, inhibition of TASK-1 current would still be expected *in vivo*. At plasma levels of 10 µM an inhibition of 20% of TASK-1 current is expected. It has to be noticed, however, that TASK-1 blockade by ranolazine is always likely to be less extensive than late I_Na_ (IC_50_ 5.9–15 µM) (Fredj et al., 2006; Antzelevitch et al., 2011) or I_Kr_ blockade (IC_50_ 8–12 µM) (Rajamani et al., 2008; Du et al., 2014). Therefore, the clinical relevance of TASK-1 blockade by ranolazine remains speculative.

We identified the TASK-1 central cavity of the inner pore as the binding site of ranolazine. This binding site overlaps with that of other both high and low affinity blockers of TASK-1 (Streit et al., 2011; Kiper et al., 2015; Schmidt et al., 2018; Wiedmann et al., 2019). The binding configuration of ranolazine within the TASK-1 inner pore is also similar to results published by Du et al. (2014) for the human Ether à go go Related Gene (hERG) channel, the recombinant equivalent of I_Kr_. In both TASK-1 and hERG, ranolazine lies high within the inner pore in a horizontal orientation below the selectivity filter where it may form hydrogen bonds with serine or threonine residues (Du et al., 2014). In both channels ranolazine can make additional hydrophobic interactions with pore lining residues (**Figures 5** and **8**). The structural basis for the higher inhibitory potency of ranolazine in hERG may result from a more optimal orientation of the pore lining side chains in hERG, especially in the inactivated state (Chen et al., 2002; Du et al., 2014). Evidence for this hypothesis comes from the observation that mutations of hERG outside the binding site that attenuate channel inactivation and influence configuration of the pore lining side chains to non-optimal arrangements, largely reduce the inhibitory potency of ranolazine without changing the binding site itself (Du et al., 2014).

The binding site is also similar to that in sodium channels, where ranolazine has been shown to interact over a larger surface area that spans from the inner pore to the side fenestration region (Fredj et al., 2006; Nguyen et al., 2019). In sodium channels, access to this binding site is most likely through the cytosolic mouth to the pore and therefore requires opening of the activation gate (Fredj et al., 2006; Wang et al., 2008; Caves et al., 2017). This mechanism provides the structural basis of the use-dependent block of sodium channels (Catterall 2012). In TASK-1, however, the inner pore region is constitutively open and therefore accessible for inhibitors. This may explain why we do not observe use-dependent block of TASK-1 by ranolazine. Another consequence of the TASK-1 drug binding site being constitutively accessible is that drug dissociation can occur at all time, whereas in sodium channels it requires the channel to be in the resting state (Antzelevitch et al., 2011). This may partly explain the higher inhibitory potency of ranolazine in sodium channels than in the constitutively open TASK-1 channel.

The side fenestrations mentioned earlier as being part of the drug binding site have recently been suggested to play an important role in K_2P_ channel pharmacology by providing an “anchor” for stable binding of inhibitors (Ramirez et al., 2017). In our T16cq6 model (where the model template is TREK-2) the side fenestrations are closed. Therefore, ranolazine is binding solely within the inner pore of TASK-1. When docking ranolazine to the T13ukm model (model template TWIK-1), where the side fenestrations are open, we observe similar binding modes as in the T16cq6 model (**Supplementary Figure S4**). However, with the side fenestrations open, the binding site indeed spans from the inner pore to the entrance of the side fenestrations, because the side chains of L122 and L239 are partly facing towards the fenestrations. Nevertheless, the significance of the side fenestrations in TASK-1 remains speculative, because the structure of TASK-1 has not been revealed yet, and the fenestration state of TASK-1 homology models is ultimately depending on the template used for homology modelling (open fenestrations in TWIK-1, closed fenestrations in TREK-2).

Single mutants F125A, Q126A, L232A and I235A showed no difference in ranolazine blockade, although being predicted to be relevant for ranolazine binding in the *in silico* docking calculations or being identified as binding site for the high affinity blockers S20951 (A1899) and AVE1231 (A293). The reason could be the relatively small size of the ranolazine molecule compared to S20951 and AVE1231. Ranolazine appears to be able to fold into different conformations and hence bind at different sites within the inner pore. The molecular interactions between ranolazine and its binding site are mostly hydrophobic interactions. Only with residues T93 and T199 it forms more stable hydrogen bonds. The ability to form hydrogen bonds appears to be impaired in channel variant L122A, possibly explaining the fact that inhibition by ranolazine is almost abolished in this single mutant.

### Study Limitations

Of note, channel variant N240A impedes ranolazine binding without being proposed to be part of the binding site in the *in silico* simulations. It remains unclear whether this is due to methodological limitations of homology modelling and docking simulations or results from interferences of the large asparagine side chain with structural domain morphology, therefore impeding the accessibility to the TASK-1 inner pore for inhibitors. The latter would be supported by the observation that movements or alterations of the M4 transmembrane domain (where N240 is located) affect channel gaiting in other K_2P_ channels (Bagriantsev et al., 2011; Lolicato et al., 2014). However, results obtained by homology modelling are naturally largely influenced by the template that is chosen for modelling. We therefore tried to assess the models quality to our best possibilities. To assess possible influences of the fenestration state of our model, we performed additional docking simulations on the T1ukm model. Of course, deeper insights in this regard would require the solvation of the TASK-1 crystal structure.

Another limitation of this study is that a direct effect of the TASK-1 mutants on channel gating and therefore possible allosteric effects on TASK-1 pharmacology cannot be ruled out. Nevertheless, measurements of an inhibitor’s potency on different central pore mutants and comparisons with *in silico* docking simulations on homology models has been a widely accepted strategy in the field thus far.

## Conclusions

This study adds to the action profile of ranolazine. We demonstrate that ranolazine is a TASK-1 inhibitor and suggest that TASK-1 inhibition may contribute to the antiarrhythmic effects of ranolazine. We propose that TASK-1 inhibition could, at least in part, explain the atrial selectivity of APD-prolongation by ranolazine. Even though the efficacy of ranolazine in AF might not yet be optimal, our findings put forward ranolazine as a prototype drug for the treatment of atrial arrhythmia because of its combined efficacy on atrial electrophysiology and lower risk for ventricular side effects.

## Data Availability Statement

The raw data supporting the conclusions of this manuscript will be made available by the authors upon reasonable request.

## Ethics Statement

The animal study was reviewed and approved by Regierungspräsidium Karlsruhe.

## Author Contributions

AR, CS, and HK conceived and designed the experiments. AR, FW, and MK carried out the experiments. AR, FW, MK, and CS contributed to the interpretation of the results. AR and CS visualized the data and wrote the manuscript. HK and CS supervised the project. All authors provided critical feedback and helped shape the research, analysis and manuscript by providing important intellectual content. Further, all persons designated as authors qualify for authorship, and all those who qualify for authorship are listed. All authors agree to be accountable for all aspects of the work in ensuring that questions related to the accuracy or integrity of any part of the work are appropriately investigated and resolved. All authors approved the final version of the manuscript.

## Funding

This study was supported in part by research grants from the University of Heidelberg Faculty of Medicine [Rahel Goitein-Straus Scholarship and Olympia-Morata Scholarship (to CS)], the German Center for Cardiovascular Research [Excellence Grant (to CS)], the German Heart Foundation/German Foundation of Heart Research [Grant F/41/15 (to CS), Grant F/35/18 (to FW and CS) and a Kaltenbach Scholarship (to AR and FW)], and the German Research Foundation [Grant SCHM 3358/1-1 (to CS)]. FW was supported by a German Cardiac Society Otto-Hess Scholarship and Research Scholarship. We acknowledge financial support by Deutsche Forschungsgemeinschaft within the funding programme Open Access Publishing, by the Baden-Württemberg Ministry of Science, Research and the Arts and by Ruprecht-Karls-Universität Heidelberg.

## Conflict of Interest

The authors declare that the research was conducted in the absence of any commercial or financial relationships that could be construed as a potential conflict of interest.
