# Supplementary material for: Antiarrhythmic Properties of Ranolazine: Inhibition of Atrial Fibrillation Associated TASK-1 Potassium Channels
Source: Front Pharmacol. 2019 Nov 26;10:1367. doi: 10.3389/fphar.2019.01367 (PMC6988797; doi:10.3389/fphar.2019.01367)
Supplement: Supplementary file 1 [file DataSheet_1.pdf]

Supplementary Material

1 Supplementary Methods

1.1 *Xenopus laevis* oocyte preparation

Xenopus laevis oocytes are one of the most commonly used expression systems for functional characterization of ion channels and have been widely used since the seventies (Gurdon et al. 1971, Dascal 1987). Their size, availability and stable expression of exogenous proteins allow for a wide range of manipulations, including electrode penetrations for low noise and long-lasting electrophysiological measurements (Lin et al. 2006). This study was carried out in accordance with the directive 2010/63/EU of the European Parliament, and the current version of the German Law on the Protection of Animals. Approval for experiments involving *Xenopus laevis* was granted by Regierungspräsidium Karlsruhe (institutional approval numbers A-38/11 and G-221/12).

Adult female *Xenopus laevis* frogs were ordered from a commercial vendor (Xenopus Express, Lyon, France). Frogs were maintained in a specific pathogen free state of the art aquarium facility with continuous water flow (Aqua Schwarz GmbH, Göttingen, Germany). Room temperature was kept at 20 °C ± 2 °C and room lighting had a light/dark cycle of 12 / 12 hours. Water in the frog housing tanks was kept at 18 °C with a maximum housing density according to directive 2010/63/EU. Frogs were fed daily with food extrudate 3590.ES.F10 (Kliba Nafag, Kaiseraugst, Switzerland). Environmental enrichment was provided with PVC pipes and huts.

Ovarian lobes were surgically removed in aseptic technique from female *Xenopus laevis* frogs anesthetized with 0.3 % ethyl 3-aminobenzoate methanesulfonate solution (Tricaine, pH 7.5, 15 °C) (Sigma Aldrich, St. Louis, MO, USA). To avoid emesis during anesthesia, frogs were not fed on the day of surgery. After surgery, the frogs were allowed to recover consciousness, followed by a recovery period of at least 30 weeks. Oocyte collection was alternated between left and right ovaries. There were no more than four procedures performed on one individual frog. After the final taking of oocytes, the anesthetized frog was killed by decerebration.

Oocytes were manually isolated under a stereomicroscope (STEMI 2000, Zeiss, Oberkochen, Germany). To remove residual connective tissue, oocytes were incubated at 18 °C for 12 h in 25 ml OR2 solution containing 96 mM NaCl, 2 mM KCl, 1 mM $MgCl_2$, and 10 mM 4-(2-hydroxyethyl)piperazine-1-ethanesulfonic acid (HEPES) (pH 7.6) substituted with 10 – 12 mg collagenase D (Roche Diagnostics, Mannheim, Germany), 125 mg bovine serum albumin (BSA) (Sigma Aldrich, St. Louis, MO, USA) and 32 mg Na_2HPO_4. Following collagenase treatment oocytes were washed in OR2 solution and stored at 18 °C in SOS solution containing 100 mM NaCl, 2 mM KCl, 1.8 mM $CaCl_2$, 1 mM $MgCl_2$, and 5 mM HEPES (pH7.6) substituted with 275 mg/l pyruvic acid and 100 mg/l gentamicin sulphate (Sigma Aldrich, St. Louis, MO, USA). For electrophysiological

recordings, cRNA (1.5 – 9 ng; 46 nl/oocyte) encoding the studied channels was injected using the Nanoject system (Drummond Scientific Company, Broomall, PA, USA).

1.2 Electrophysiology

Two-electrode voltage clamp (TEVC) recordings from *Xenopus laevis* oocytes were performed one to three days after cRNA injection. Pipettes were pulled from 1 mm borosilicate glass tubes (GB 100F-10, Science Products, Hofheim, Germany) using a P-87 micropipette puller (Sutter Instruments, Novato, CA, USA) and had tip resistances of 0.5 – 1.5 MΩ. Pipettes were filled with internal solution containing 96 mM NaCl, 3 mM KCl, 1.1 mM $CaCl_2$, 1 mM $MgCl_2$, and 5 mM HEPES. Experiments were performed under constant perfusion by a gravity-driven perfusion system. The standard extracellular bath solution contained 96 mM NaCl, 4 mM KCl, 1.1 mM $CaCl_2$, 1 mM $MgCl_2$, and 5 mM HEPES. The pH was adjusted with NaOH to pH 7.4 except for measurements of TALK-1 and TALK-2 currents (pH 8.5). Macroscopic currents were recorded using an OC-725C Oocyte Clamp amplifier (Warner Instruments, Hamden, CT, USA), a Digidata 1322A Series (Axon Instruments, Foster City, CA, USA) and pClamp 10 software (Molecular Devices, San José, CA, USA). Data were sampled at 2 kHz and low-pass filtered at 1 kHz. From a holding potential of −80 mV, test pulses were applied for 500 ms to voltages between −140 and +60 mV in 20 mV increments (0.2 Hz) unless stated otherwise. Current amplitude was quantified at the end of each 500 ms test pulse. Current recordings from CHO cells were carried out using the whole-cell patch clamp technique with an Axopatch 200B amplifier (Axon Instruments, Foster City, CA, USA), an Axon Digidata 1550B series (Axon Instruments, Foster City, CA, USA), and pClamp 10 software (Molecular Devices, San José, CA, USA). Glass pipettes (1B120F-4; World Precision Instruments, Berlin, Germany) with tip resistances ranging from 2 to 5 MΩ were filled with the following solution: 100 mM K-aspartate, 20 mM KCl, 2 mM $MgCl_2$, 1 mM $CaCl_2$, 10 mM EGTA, 2 mM Na_2ATP, and 10 mM HEPES (pH 7.2). The extracellular bath solution contained 140 mM NaCl, 5 mM KCl, 1 mM $MgCl_2$, 1.8 mM $CaCl_2$, 10 mM HEPES, and 10 mM glucose (pH 7.4). Seal resistances yielded 4 – 8 GΩ. Series resistance and cell capacitance were compensated. Data were not corrected for liquid junction potentials. Membrane currents were evoked by application of voltage steps between −80 and +60 mV in 10 mV-increments (duration 500 ms; holding potential −80 mV). All experiments were carried out at room temperature (20 – 22 °C). Leak currents were not subtracted.

1.3 Molecular docking calculations

Molecular docking calculations were performed using AutoDock Vina (Trott and Olson 2010). Polar hydrogens and Kollmann charges were added using AutoDockTools 1.5.6 (Morris et al. 2009). The grid box was a cubic box with an axial length of 35 Å and was centered to the intracellular central channel cavity, where in previous studies TASK-1 inhibitors were identified to bind (Streit et al. 2011, Chokshi et al. 2015, Wiedmann et al. 2019). The Ranolazine ligand was obtained from PubChem database (CID: 56959) (Kim et al. 2016). Non-polar hydrogens were automatically merged by AutoDockTools, the central atom was detected for use as the root, and 11 rotatable bonds were identified. Docking simulations were performed with potassium ions in positions S1 and S3 or S2 and S4 within the selectivity filter of TASK-1 (see Figure 5 of main manuscript). Each calculation yielded 10 ranked docking poses.

2 Supplementary Results and Discussion

2.1 MolProbidity assessment of TASK-1 homology models

Four TASK-1 homology models were built based on the structures of TWIK-1 (protein data bank (PDB) ID: 3UMK) (Miller and Long 2012), TREK-1 (PDB ID: 6CQ6) (Lolicato et al. 2017), TREK-2 (PDB ID: 4XDL) (Dong et al. 2015) and TRAAK (PDB ID: 4RUE) (Lolicato et al. 2014) (Figure S1). They were named according to their respective reference structure: *T16cq6* (TASK-1 model based on the crystal structure of TREK-1), *T13umk* (based on TWIK-1), *T14rue* (based on TRAAK), and *T14xdl* (based on TREK-2). The MolProbidity assessment (Davis et al. 2007) yielded *T16cq6* as being the most appropriate model (Figure S2). Therefore this model was used for *in silico* docking simulations.

2.2 Mutations within the TASK-1 inner pore region results in an altered binding site and protein-ligand interaction profile of ranolazine and TASK-1

To study ranolazine binding to TASK-1 mutant variants in detail, *in silico* docking simulations were performed on the single mutants T93A, L122A, F125A, T199A, L232A and I235A that had been shown to be most relevant for ranolazine binding in the initial WT docking simulation. Figure S3 provides an overview about protein-ligand interaction profiles of ranolazine and the different TASK-1 variants in comparison to WT TASK-1. Ranolazine was still able to bind at the inner pore of the TASK-1 mutant variants. However, the protein-ligand interaction profiles were altered. The graph illustrates that ranolazine binding is particularly impaired in mutant L122A. The amount of hydrophobic interactions of ranolazine and the inner pore does not significantly vary among the other TASK-1 variants. However, the ability to form relatively stable hydrogen bonds is significantly reduced in T93A, L122A and T199A (see also Figure 7 in main manuscript).

3 Supplementary Figures

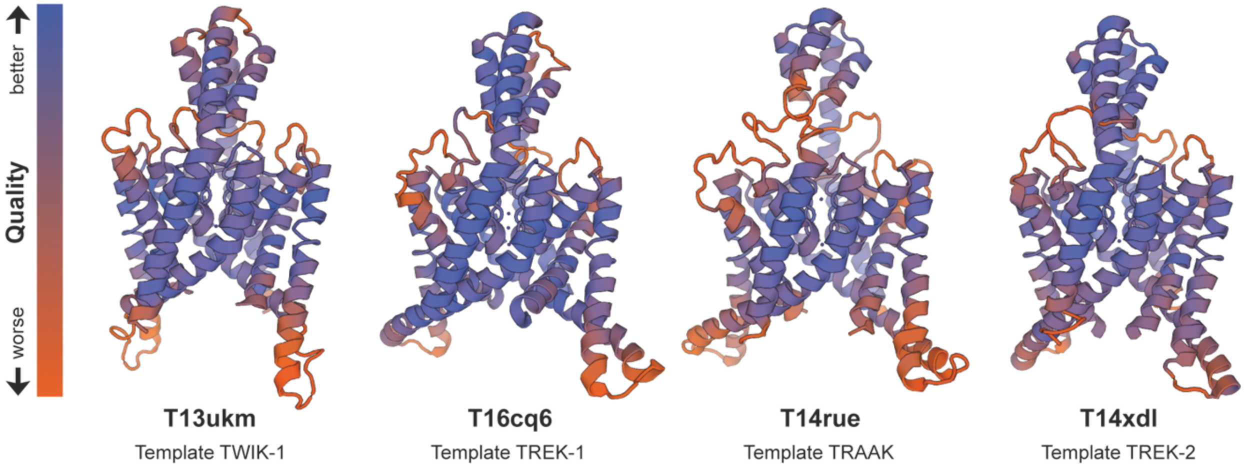

Figure S1 Illustrations of the four generated TASK-1 homology models. The local QMEAN (an estimate of the local model quality calculated by SWISS-MODEL) is color coded with blue reflecting a good local model quality and orange reflecting a bad local quality.

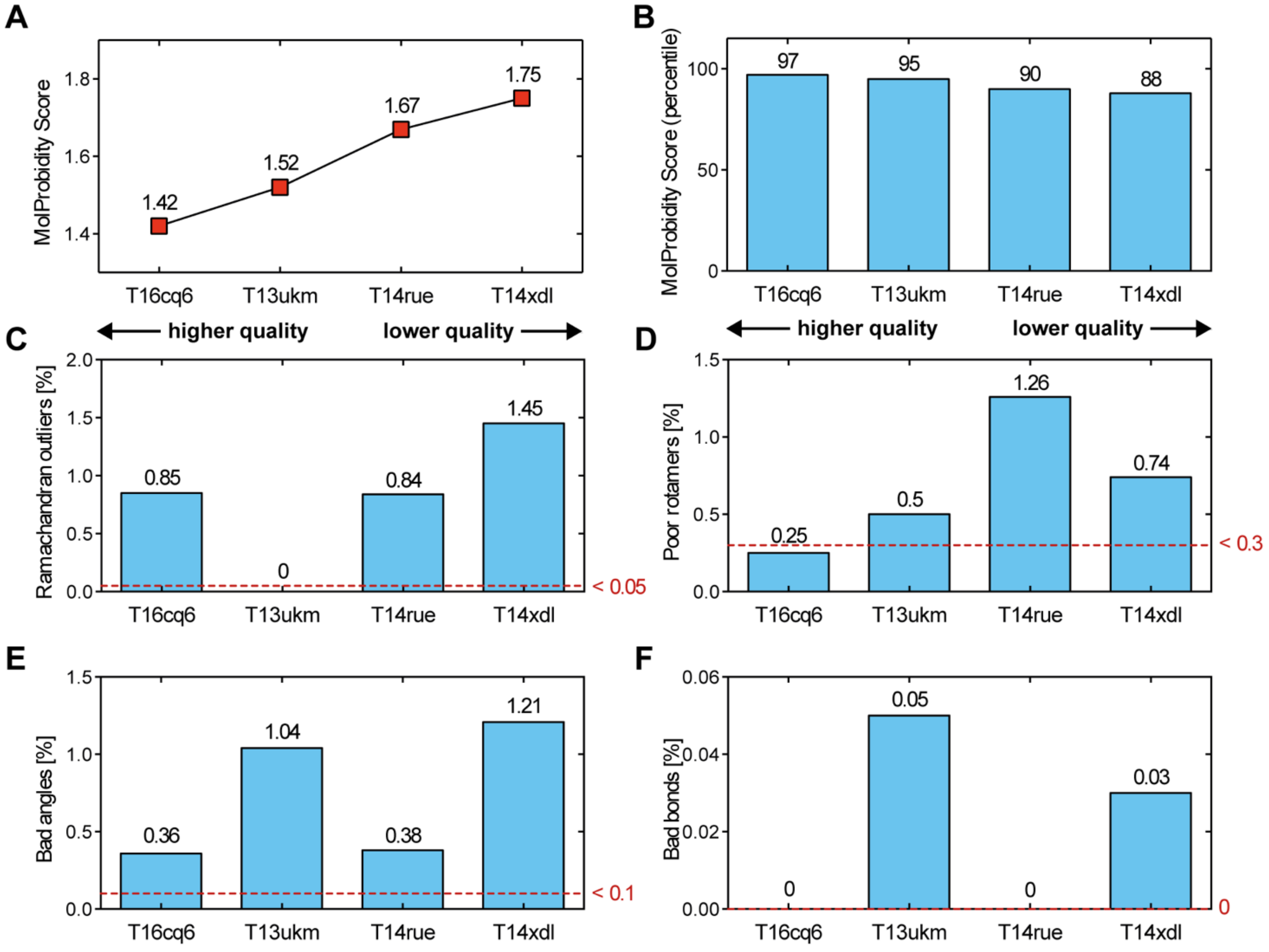

Figure S2 MolProbidity Assessment of TASK-1 homology models T16cq6 (TASK-1 model based on the crystal structure of TREK-1; Protein Data Bank (PDB) ID: 6CQ6), T13umk (based on TWIK-1; PDB ID: 3UMK), T14rue (based on TRAAK; PDB ID: 4RUE), and T14xdl (based on TREK-2; PDB ID: 4XDL). (A, B) The MolProbidity Score is given as a numerical value (A) and as the respective percentile (B). The score summarizes different aspects of all-atom contacts and protein geometry that are being assessed during the MolProbidity analysis. Some of these aspects are reported in (C – F) (Davis et al. 2007). A model with a lower MolProbidity score is considered a more accurate model. (C) The percentage of outliers in the Ramachandran analysis (Ramachandran et al. 1963) is given for the different models. A value of < 0.05 is considered favorable. (D) Proportion of poor rotamers within the respective model. A value of < 0.3 is preferable. (E) Proportion of bad angles within the respective model. A value of < 0.1 is preferable. (F) Proportion of bad bonds within the respective model. A value of 0 is preferable.

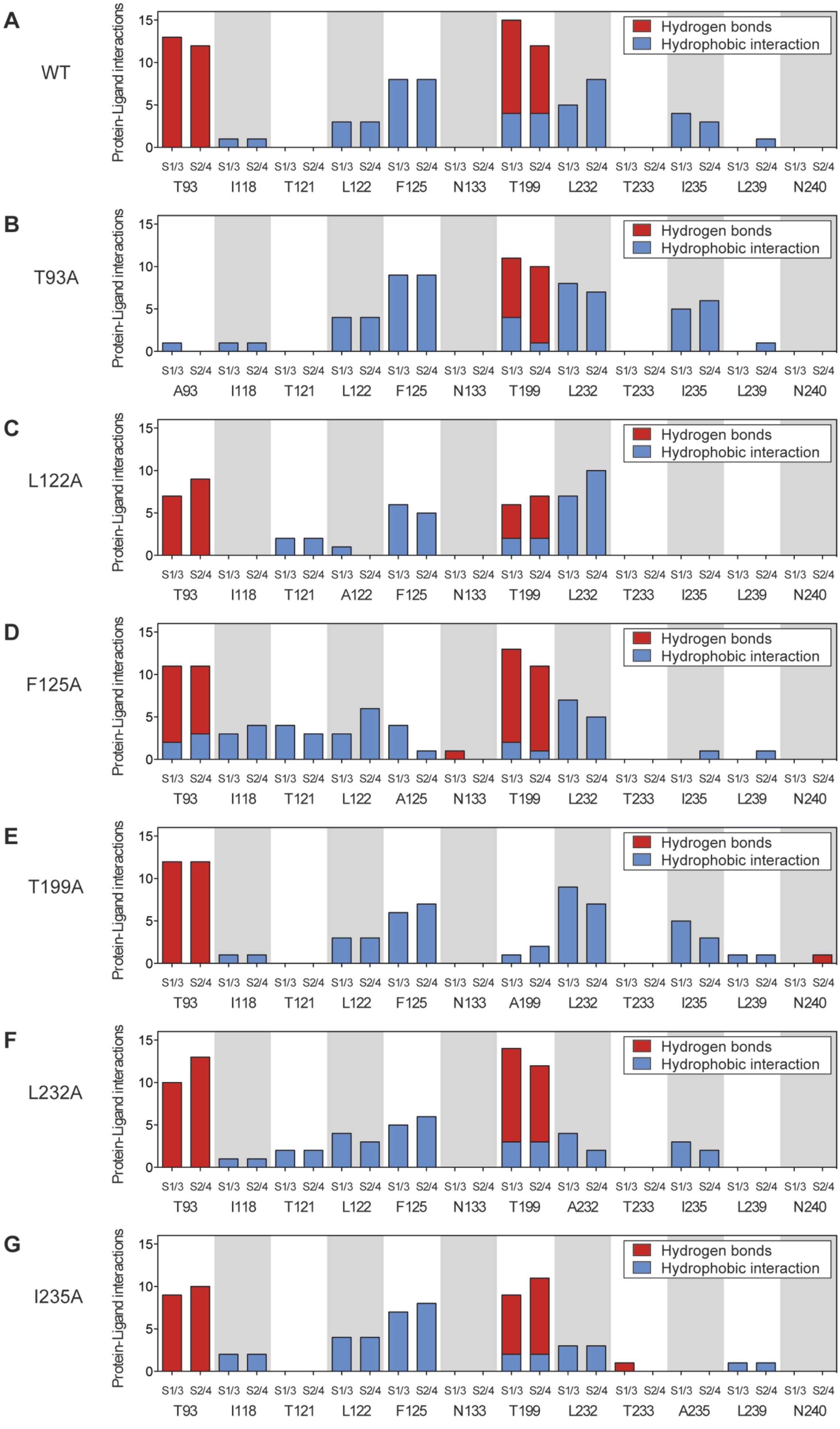
A
WT
Protein-Ligand interactions
15
10
5
0
Hydrogen bonds
Hydrophobic interaction
S1/3 S2/4 S1/3 S2/4 S1/3 S2/4 S1/3 S2/4 S1/3 S2/4 S1/3 S2/4 S1/3 S2/4 S1/3 S2/4 S1/3 S2/4 S1/3 S2/4 S1/3 S2/4 S1/3 S2/4
T93 I118 T121 L122 F125 N133 T199 L232 T233 I235 L239 N240
B
T93A
Protein-Ligand interactions
Hydrogen bonds
Hydrophobic interaction
A93 I118 T121 L122 F125 N133 T199 L232 T233 I235 L239 N240
C
L122A
Protein-Ligand interactions
Hydrogen bonds
Hydrophobic interaction
T93 I118 T121 A122 F125 N133 T199 L232 T233 I235 L239 N240
D
F125A
Protein-Ligand interactions
Hydrogen bonds
Hydrophobic interaction
T93 I118 T121 L122 A125 N133 T199 L232 T233 I235 L239 N240
E
T199A
Protein-Ligand interactions
Hydrogen bonds
Hydrophobic interaction
T93 I118 T121 L122 F125 N133 A199 L232 T233 I235 L239 N240
F
L232A
Protein-Ligand interactions
Hydrogen bonds
Hydrophobic interaction
T93 I118 T121 L122 F125 N133 T199 A232 T233 I235 L239 N240
G
I235A
Protein-Ligand interactions
Hydrogen bonds
Hydrophobic interaction
T93 I118 T121 L122 F125 N133 T199 L232 T233 A235 L239 N240

Figure S3 *(Figure on previous page)* Summary of protein-ligand interactions of all calculated docking poses of ranolazine at the inner pore of the wild type (WT) TASK-1 model and the different mutant variants. (A – G) The amount and character of interactions is displayed for the individual amino acid residues that interact with ranolazine. See also Figure 7 in main manuscript.

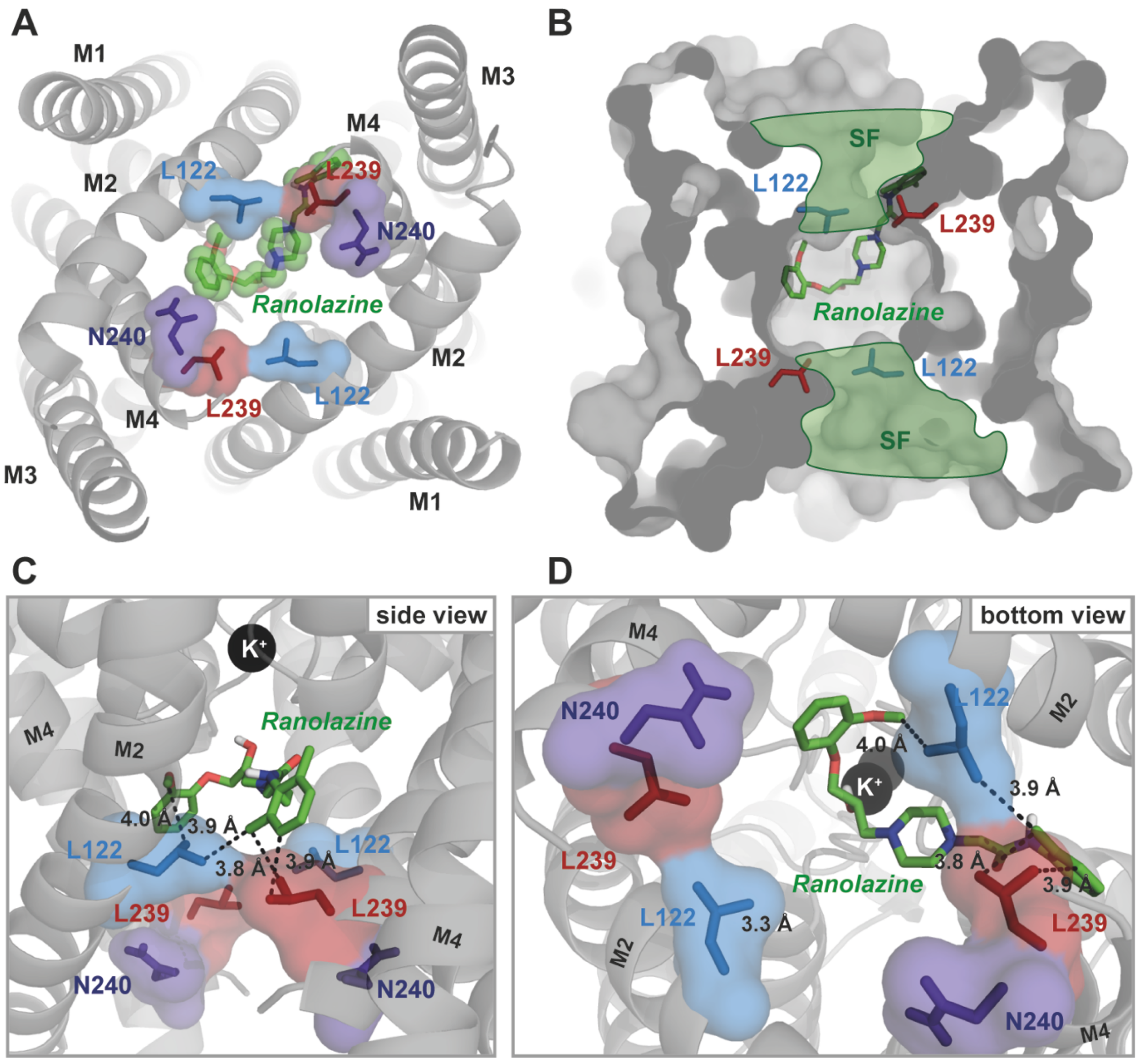

Figure S4 Ranolazine binding to T13ukm homology model. Ranolazine binds at the central cavity of the inner pore at the entrance into the side fenestrations of T13ukm. (A) View from inside the cell into the central cavity. Ranolazine binds in close proximity to L122 and L239 occluding the lumen of the inner pore and reaching into the side fenestrations. (B) Same view as in (A) but with TASK-1 surface representation illustrating the open side fenestrations. (C) Zoom into the central cavity illustrating the binding mode and the distance to the residues identified as binding site in the mutagenesis screen. Note that ranolazine does not directly interact with N240. (D) Similar zoom as in (C) but from inside the cell.

4 References

Chokshi, R. H., Larsen, A. T., Bhayana, B. and Cotten, J. F. (2015). "Breathing Stimulant Compounds Inhibit TASK-3 Potassium Channel Function Likely by Binding at a Common Site in the Channel Pore." Mol Pharmacol **88**(5): 926-934.

Dascal, N. (1987). "The use of Xenopus oocytes for the study of ion channels." CRC Crit Rev Biochem **22**(4): 317-387.

Davis, I. W., Leaver-Fay, A., Chen, V. B., Block, J. N., Kapral, G. J., Wang, X., et al. (2007). "MolProbity: all-atom contacts and structure validation for proteins and nucleic acids." Nucleic Acids Res **35**(Web Server issue): W375-383.

Dong, Y. Y., Pike, A. C., Mackenzie, A., McClenaghan, C., Aryal, P., Dong, L., et al. (2015). "K2P channel gating mechanisms revealed by structures of TREK-2 and a complex with Prozac." Science **347**(6227): 1256-1259.

Gurdon, J. B., Lane, C. D., Woodland, H. R. and Marbaix, G. (1971). "Use of frog eggs and oocytes for the study of messenger RNA and its translation in living cells." Nature **233**(5316): 177-182.

Kim, S., Thiessen, P. A., Bolton, E. E., Chen, J., Fu, G., Gindulyte, A., et al. (2016). "PubChem Substance and Compound databases." Nucleic Acids Res **44**(D1): D1202-1213.

Lin, B. R., Gierasch, L. M., Jiang, C. and Tai, P. C. (2006). "Electrophysiological studies in Xenopus oocytes for the opening of Escherichia coli SecA-dependent protein-conducting channels." J Membr Biol **214**(2): 103-113.

Lolicato, M., Arrigoni, C., Mori, T., Sekioka, Y., Bryant, C., Clark, K. A., et al. (2017). "K2P2.1 (TREK-1)-activator complexes reveal a cryptic selectivity filter binding site." Nature **547**(7663): 364-368.

Lolicato, M., Riegelhaupt, P. M., Arrigoni, C., Clark, K. A. and Minor, D. L., Jr. (2014). "Transmembrane helix straightening and buckling underlies activation of mechanosensitive and thermosensitive K(2P) channels." Neuron **84**(6): 1198-1212.

Miller, A. N. and Long, S. B. (2012). "Crystal structure of the human two-pore domain potassium channel K2P1." Science **335**(6067): 432-436.

Morris, G. M., Huey, R., Lindstrom, W., Sanner, M. F., Belew, R. K., Goodsell, D. S., et al. (2009). "AutoDock4 and AutoDockTools4: Automated docking with selective receptor flexibility." J Comput Chem **30**(16): 2785-2791.

Ramachandran, G. N., Ramakrishnan, C. and Sasisekharan, V. (1963). "Stereochemistry of polypeptide chain configurations." J Mol Biol **7**: 95-99.

Streit, A. K., Netter, M. F., Kempf, F., Walecki, M., Rinne, S., Bollepalli, M. K., et al. (2011). "A specific two-pore domain potassium channel blocker defines the structure of the TASK-1 open pore." J Biol Chem **286**(16): 13977-13984.

Trott, O. and Olson, A. J. (2010). "AutoDock Vina: improving the speed and accuracy of docking with a new scoring function, efficient optimization, and multithreading." J Comput Chem **31**(2): 455-461.

Wiedmann, F., Kiper, A. K., Bedoya, M., Ratte, A., Rinne, S., Kraft, M., et al. (2019). "Identification of the A293 (AVE1231) Binding Site in the Cardiac Two-Pore-Domain Potassium Channel TASK-1:

a Common Low Affinity Antiarrhythmic Drug Binding Site." Cell Physiol Biochem **52**(5): 1223-1235.
